# Green Rust: The Simple Organizing ‘Seed’ of All Life?

**DOI:** 10.3390/life8030035

**Published:** 2018-08-27

**Authors:** Michael J. Russell

**Affiliations:** Planetary Chemistry and Astrobiology, Jet Propulsion Laboratory, California Institute of Technology, Pasadena, CA 91109-8099, USA; michael.j.russell@jpl.nasa.gov

**Keywords:** Hadean, carbonic ocean, mantle plumes, banded iron formation, green rust, submarine alkaline vents, emergence of life

## Abstract

Korenaga and coworkers presented evidence to suggest that the Earth’s mantle was dry and water filled the ocean to twice its present volume 4.3 billion years ago. Carbon dioxide was constantly exhaled during the mafic to ultramafic volcanic activity associated with magmatic plumes that produced the thick, dense, and relatively stable oceanic crust. In that setting, two distinct and major types of sub-marine hydrothermal vents were active: ~400 °C acidic springs, whose effluents bore vast quantities of iron into the ocean, and ~120 °C, highly alkaline, and reduced vents exhaling from the cooler, serpentinizing crust some distance from the heads of the plumes. When encountering the alkaline effluents, the iron from the plume head vents precipitated out, forming mounds likely surrounded by voluminous exhalative deposits similar to the banded iron formations known from the Archean. These mounds and the surrounding sediments, comprised micro or nano-crysts of the variable valence Fe^II^/Fe^III^ oxyhydroxide known as green rust. The precipitation of green rust, along with subsidiary iron sulfides and minor concentrations of nickel, cobalt, and molybdenum in the environment at the alkaline springs, may have established both the key bio-syntonic disequilibria and the means to properly make use of them—the elements needed to effect the essential inanimate-to-animate transitions that launched life. Specifically, in the submarine alkaline vent model for the emergence of life, it is first suggested that the redox-flexible green rust micro- and nano-crysts spontaneously precipitated to form barriers to the complete mixing of carbonic ocean and alkaline hydrothermal fluids. These barriers created and maintained steep ionic disequilibria. Second, the hydrous interlayers of green rust acted as engines that were powered by those ionic disequilibria and drove essential endergonic reactions. There, aided by sulfides and trace elements acting as catalytic promoters and electron transfer agents, nitrate could be reduced to ammonia and carbon dioxide to formate, while methane may have been oxidized to methyl and formyl groups. Acetate and higher carboxylic acids could then have been produced from these C1 molecules and aminated to amino acids, and thence oligomerized to offer peptide nests to phosphate and iron sulfides, and secreted to form primitive amyloid-bounded structures, leading conceivably to protocells.

*The most important problem of synthetic biology is…the reduction of carbonic acid.*

*Without the idea of spontaneous generation and a physical theory of life, the doctrine of evolution is a mutilated hypothesis without unity or cohesion.*

-Leduc [1]

## 1. Introduction

Chemistry unbridled could not have led to life [1,2,3,4]. Redox and pH gradients were required together with ‘engines of disequilibria conversion’ to allow these gradients to do the work of surmounting the steep endergonic barriers encountered by C1 molecules so that they might have reacted and complexified along the pathways and, eventually, around the autocatalytic cycles of life, just as the autotrophs feeding the base of the food web do today [5]. But what were the materials and what was the environment that could have allowed these gradients to be used to drive the key endergonic processes needed to launch life? In our original alkaline hydrothermal vent (AHV) model, we suggested that life emerged at an alkaline hydrothermal spring on meeting the carbonic Hadean Ocean [5]. At the macro level, the spontaneously precipitated sulfides and hydroxides could have induced just the redox and pH gradients, and with the same polarities as extant life [5,6]. Additionally, the precipitate mounds could have provided the conversion engines specific to the transformations that needed to occur in the ambient environment, sufficient to drive life’s emergence [3,4,7]. However, it became clear that mere porous cavities could not provide the low water activities, the molecular crowding, and pumps beyond those merely osmotic, to meet the requirements of an autotrophic metabolism, let alone its need for adaption, guidance, and waste disposal [7]. Moreover, a mineral seed has struck many as lacking the versatility of organic molecules, whatever their provenance. So, when Arrhenius [8] also called upon a crystal as being a host mineral of biopoesis, it was not obvious then that a genuine epiphany had been realized. Arrhenius’ crystal group of choice was the Doppelschichtstrukturen mit brucitähnlichen, first described by Allmann [9,10]. These double layer hydroxides (DLH, though generally and mistakenly referred to as the layered double hydroxides, LDH) may be derived from the brucite (Mg[OH]_2_) structure, especially significant in that brucite itself is often the first mineral to be precipitated at submarine alkaline hydrothermal vents today [11,12,13]. In the DLH proper, two-thirds of the cations are divalent (e.g., Mg^2+^, Fe^2+^, Ni^2+^, Zn^2+^, Ca^2+^, Co^2+^, and Mn^2+^), whereas every third cation carries a +3 charge (e.g., Al^3+^, Fe^3+^, Cr^3+^, Mn^3+^, Ni^3+^, and Co^3+^). One such DLH, green rust (~Fe^II^_4_Fe^III^_2_[OH]_12_CO_3_·3H_2_O), struck Arrhenius [8] as a particularly good candidate to consider in terms of life’s emergence. It is pliant, contains hydrous interlayers that readily persorb anions, and is both responsive and resilient to strong variations in pH and redox. We might think of these interlayers as the viscous innards, or even as proto-cytoplasm within the spontaneously precipitated and rechargeable green rust. Green rust, along with the subordinate iron sulfide mackinawite ([Fe>Ni]S) are proposed to have functioned as electrochemical nano-engines acting to convert the imposed external proton and redox disequilibria into the internal disequilibria necessary to bring life into being [4,7]. These engines might effect free energy conversions of various types driven by Brownian motion with built-in escapement mechanisms that prevent back reactions [3,4,14]. It is in this light that discerning such recognizable mineral precursors in the geologic record should be possible, to explain the autogenic emergence of metabolic pathways and cycles supplied from the simplest of carbon substrates, carbon dioxide, formate, and methane [7,14,15]. In the AHV model, the complex molecular engines mediating present-day electron transfer and conversions are a result of evolution from these abiotic mineral ancestors [3,4,7,14].

Thus, the contribution of this study is suggesting how green rust (~[Fe^2+^_6x_Fe^3+^_6(1−x)_O_12_H_2(7−3x)_]^2+^·[CO^2−^_3_·3H_2_O]^2−^), nickel-rich mackinawite ([Fe>Ni]S), greigite (Fe_5_NiS_8_), violarite (Fe_2_Ni_4_S_8_), and possibly tochilinite (FeS[Mg,Fe^2+^][OH]_2_) precipitated as the first response to the alkaline vent-versus-ocean interfacing, may have been co-opted, adapted, and systematized as nano-engines and catalysts at a Hadean submarine alkaline mound, enabling the emergence of life [4,16]. That green rust is capable of conformational changes during oxidative-reductive and protonation-deprotonation oscillations lends itself to such investigations [8,17,18,19,20]. We also speculate on the crucial steps taken toward what must have been the break-out metabolism and an organic takeover of the mineral precursor disequilibrium or ‘free energy’ converters, as they acquired organic frameworks in a process that we have called ‘minerobiolization’. We then review the status of efforts to test these and related ideas related to the issue and conclude with a rather long list of falsifiable predictions of the submarine AHV model as it now stands. The transition from inorganic-enabled toward partly organic-enabled molecular mechanisms was, we think, just the very first of many subsequent evolutionary steps taken through the adaption of a chance function derived in one context for use in another. In the text, references to analogous modern mechanisms to those considered to have first begat life can be thought of as the direct legacies from, and as being broadly homologous with, their mineral progenitors. With these assumptions in mind, we suggest a testable model whereby the first and simplest pathways and enzymatic mechanisms are assumed to reflect on early beginnings and availabilities of disequilibria, mechanisms for their conversion, as well as on the trace metals and phosphate that contributed to these processes both then and now.

## 2. Model Assumptions

The submarine AHV model for life’s emergence focusses on the interaction between C1 molecules and proton and electron gradients, acting across membranes to drive autogenesis [6,16,20,21,22,23]. The iron oxyhydroxides and sulfides comprising the membranes act as disequilibrium converters [4,7,14,16]. However, these and several other key aspects of the AHV theory need experimental testing, particularly at pressures high enough (~10 bars) to keep hydrogen, methane and CO_2_ in sterile solution. Lane’s group had some success testing this autotrophic (autogenic) hydrothermal model for the emergence of life by attempting to reproduce molecules of the reductive acetyl coenzyme-A pathway (rAcCoA) [24,25,26,27,28,29]. This group tested an abiotic CO_2_-fixing pathway employing a rig that models the submarine alkaline vent, whereby CO_2_ dissolved in an acidulous ocean analog on one side of an FeS-bearing membrane is juxtaposed with an alkaline hydrothermal solution on the other [24]. An endergonic reaction was expected to be driven by the protons pent up in the ocean (the original and natural proton motive force) as they permeated the iron sulfide chimney toward the alkaline interior along a single pathway [24,29,30]. Yields of CO_2_ reduction products in these experiments were ~50 µM formate along with a variable and inconsistent formaldehyde concentration of around 100 nM [31]. The authors speculated that the formaldehyde product should be enough to initiate the formose reaction and thereby produce various sugars. However, formaldehyde is a significant contaminant in buildings and laboratories, which might explain the non-reproducibility of their results and give pause for thought [32,33].

Nakamura and colleagues also provided partial experimental support for the AHV theory [34]. Using iron-nickel sulfides, they demonstrated the electro-catalytic reduction of CO_2_, whereby the 3–4 pH units operating as the putative proton motive force (PMF) is converted to the ~200 mV over-potential required in the natural reduction [34]. Yamaguchi et al. cautioned that the PMF is rapidly expended through the reduction of H^+^, unless amines are available to improve the Faradaic efficiency for CO_2_ reduction [34,35,36,37,38]. Rapid aminations of pyruvate to alanine have been demonstrated, and this amino acid would, through its amino site, substantially improve electron transfer reactions and the yield of reduced carbon species, and simultaneously increase the durability of the membrane [22,34,35,36,37,38,39]. Much of any H_2_ generated in these conditions would be absorbed in the sulfides that could act as H_2_ stores and—through the re-reduction of the greigite to mackinawite—induce a homeostatic pH regulating mechanism [40,41]. The two products of these electrochemical reductions are CO and CH_4_ [34]. Beyond carbon monoxide, no metastable intermediates such as formaldehyde or a methyl group, along which a metabolic pathway might be forged, have been recorded [34].

More in line with the AHVT, an early experiment by Huber and Wächtershäuser demonstrated the production of thioacetate directly from carbon monoxide using iron and/or nickel as catalysts in neutral and alkaline conditions [42]. Their experiment called for an activated-methyl group, methane thiol, generated from CO_2_ and H_2_S or FeS/HCl and H_2_S—an ostensibly autotrophic reaction [43,44]. However, yields of the methane thiol were limited to 0.1% of the H_2_S feed, the initial concentration of which was ~1 mM/L at most, suggestive of a ≤1 μM/L thiol concentration—less than one ten thousandth of the concentration of CO_2_ around the vent [43,44,45,46,47]. That this experiment requires repeating is made the clearer by the fact that thiols are, against expectation, seemingly absent from submarine hot springs [48]. Nevertheless, these experiments do partially address Luduc’s assertion that, “The most important problem of synthetic biology is not so much the synthesis of the albuminoids as the reduction of carbonic acid”—an argument echoed by the mineralogist Goldschmidt, and provided a biochemical foundation by Fuchs [1,26,27,49,50].

**Figure 1:**
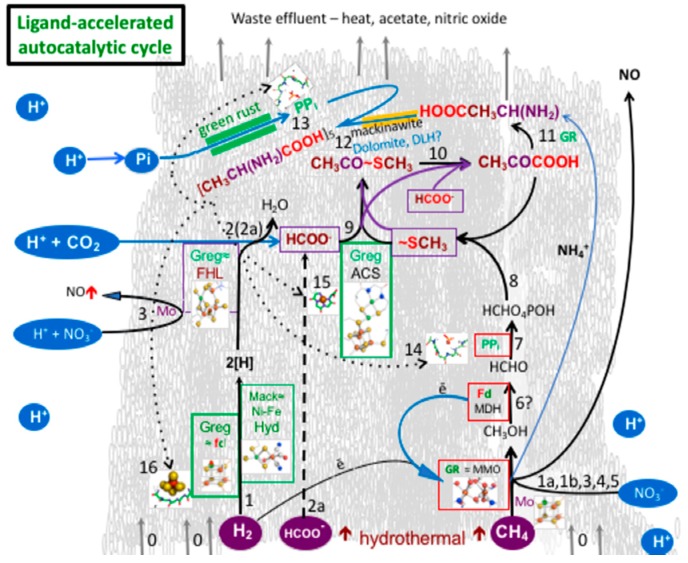
The proposed virtuous circle sketched here over a backdrop of a putative submarine green rust-mackinawite-greigite mound is intended to identify envisioned reactions demonstrated, or yet to be investigated in the lab—steps toward the first ligand-accelerated autocatalytic cycle (numbered reactions are specified in Table 1). It models denitrifying methanotrophic acetogenesis as the proposed pathway to the emergence of life in a submarine alkaline hydrothermal mound [54,55]. This model was conceived partly as a response to the generally trivial and intermittent yields of methane in our hydrothermal experiments [60]. Note, given the high kinetic barrier to the reduction of carbon dioxide, a short cut to formate may have been initially offered by the serpentinization reaction [60]. As the main drivers to the overall process are the pH and redox vectors (likely involving molybdenum as an electron bifurcator) operating across the inorganic membrane, we predict that green rust will prove to act as a general redox and pH disequilibria-converting engine in, and comprising, the membrane, supported by FeNi-sulfide catalysis and electron transfer [16,17,29,54,61,62,63,64,65,66,67,68,69,70]. The conditions responsible for these putative steps were probably localized somewhere on the margins of the mound and are only separated on this sketch for clarity.

Thus, in the face of these results and speculations, it might seem perverse to instead suggest a two-route autogenic pathway to the metastable intermediates of life. However, as the submarine alkaline vent environment offers millimolar-levels of both the fully-reduced carbon end member CH_4_, as well as the stable fully-oxidized carbon end member CO_2_, we formulated a hypothesis involving these feeds from either end of the full C1 redox span to produce activated acetate, the target molecule of the first step to metabolism [51,52,53,54]. This two-path model to activated acetate—an alternative possibility yet to be experimentally tested—has been termed ‘denitrifying methanotrophic acetogenesis’ [54]. In this model, CO_2_ is reduced to formate or CO with electrons either initially provided by oxidation of Fe(II), as in the experiments mentioned above, or by a reversal of the formate hydrogen lyase reaction [17]. At the same time, the hydrothermal CH_4_ is converted to a methyl group in complex reactions involving molybdenum in a 2-electron redox cluster that has its electrons bifurcate: one to the high electron nitrite acceptor generated in green rust, converting it to nitric oxide; and the other, now more strongly-reducing electron, accepted by a greigite (Fe_5_NiS_8_) cluster (cf. CO-dehydrogenase) [54,55]. The oxygen atom of the nitric oxide is activated by the high potential electron from the molybdenum center, which now has the power to oxidize the methane to a methyl group [54]. The two lost electrons are replaced by those released on the oxidation of hydrothermal hydrogen at a NiFe (Ni-bearing greigite) center acting as a hydrogenase. The resulting methyl group is further oxidized to a formyl group and then sulfidized to methane thiol, the two uniting to form the activated acetate as in the Huber-Wächtershäuser experiment [17,42,54]. The electron acceptors in this modified AVT are nitrate and nitrite (the first ‘breathing’ entities of life), derived from the nitrogen oxides produced mainly through cloud-to-cloud lightning and thence rained out into the Hadean ocean [55,56,57,58]. Eventually reaching micromolar concentrations, this oxidant, along with ≤350 mM of carbon dioxide, are carbureted to the mound’s margins through secondary convection and entrainment (Figure 1, Table 1) [59]. But how does such a scenario fit with what we now know of Hadean times?

## 3. The Hadean Water World

Prior to the advent of plate tectonics and following a rapid rain-out at ~4.4 Ga, about two present-day ocean volumes likely enveloped an entirely submerged mafic Hadean crust comprising extensive igneous provinces fed by large mantle plumes [119,120,121,122,123,124,125,126,127]. Judging from a modern example, the Ontong Java Plateau (that portion distant from the collision zone with the Solomon Island Arc), Hadean crust would have lain several kilometers below sea level, well out of the way of the so-called Late Heavy Bombardment [128,129,130,131]. Moreover, early Hadean zircons also suggest derivation from a strictly chondritic (i.e., “primitive”) magma reservoir through the heat-pipe tectonics of mantle plumes [131,132]. These zircons have none of the indicators for fractionation that might otherwise suggest the development of an emergent Hadean continental crust [133,134]. There really was no fresh water available.

Furthermore, conditions at the Hadean ocean surface were certainly less than clement. The length of the day was shorter, the ultraviolet (UV) flux was destructive to molecules of any length or intricacy, the moon was close, the tides were 20 times stronger, and the weather was an unchecked roiling maelstrom (Figure 1) [135,136]. Thus, there were no candidate land-based ‘birthing’ pools to periodically dry out and drive the polymerization of in-falling organic compounds to produce, at best, a picomolar organic soup, which would be of doubtful nutritious value. Such conditions lay nearly a billion years into the future, well after life’s onset, and are irrelevant to the emergence of life [137,138,139].

Extrapolating backward from early Archean exposures in Greenland, the Hadean ocean crust of this water-world was likely thick, relatively cool, and covered in carbonate green rust (~[Fe^2+^_6x_Fe^3+^_6(1−x)_O_12_H_2(7−3x)_]^2+^.[CO^2−^_3_.3H_2_O]^2−^) and silica gels derived from a myriad of sulfate-poor acidic ~400 °C hydrothermal springs [140,141,142,143,144,145,146,147,148,149]. These very hot springs were driven by heat from the mantle plumes and bore many tens of mM of iron into the carbonic Hadean ocean [142]. The pH of this ocean, as back-extrapolated and assuming equilibrium with CO_2_, has generally been over-estimated, a consequence of failing to factor in the 50-fold supersaturation of iron in carbonic fluid [143]. However, empirical evidence from iron-rich carbonic lakes in Cameroon suggest instead a pH of 5 to 5.5 [144,145]. Green rust would have precipitated from this acidulous ocean on meeting alkaline hydrothermal fluids, mainly produced by unforced open convection [16,30,46,47,148,149,150]. On burial, this green rust would have converted to hematite and magnetite constituting the thinly laminated banded iron formations (BIFs), comparable to those we see today [8,150]. With a specific gravity of around 5 and interlayered with basalt/komatiite and chert with specific gravities of ~2.9 and 2.5, respectively, the Hadean crust was doomed to founder (rather than subduct) back into the hot, dry mantle, thereby explaining its absence [140].

## 4. The Precipitate Mound at the Submarine Alkaline Vent

As noted, green rust would have been precipitated from iron derived ultimately from high temperature acidic springs [142]. This iron remained supersaturated until interfacing alkaline fluids, although some iron was likely lost to the downdrafts of neutral moderate temperature hydrothermal systems [46,148]. The main source of such alkaline waters would have been “Lost City-type” moderate temperature alkaline springs [5,11,12,13]. Subsidiary hydrothermal mackinawite [Fe>Ni]S, greigite ~Fe_5_NiS_8_), amorphous silica (SiO_2_), and phosphates (~Fe^II^_3_[PO_4_]_2_·8H_2_O) along with a few scattered grains of molybdenite, were likely to have been co-precipitates (Table 1, Figure 1) [22,30,35,45,146,151,152,153,154,155,156,157,158,159,160,161].

**Figure 2:**
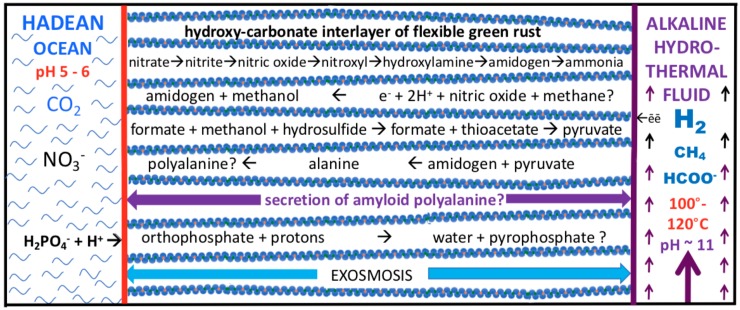
Diagram of some of the feed molecules and expected products driven by redox and pH (electron and proton) gradients as mediated within the nano-galleries of green rusts situated in, and comprising, a barrier between alkaline hydrothermal solutions and the carbonic Hadean Ocean. See text, Figure 1 and Table 1. Exosmosis, yet to be demonstrated, would obviate the need for wetting and drying cycles. Not to scale.

At alkaline hydrothermal vents in today’s oceans, brucite (Mg[OH]_2_)—a single layer hydroxide—is an early precipitate [11,12,13]. Such a wet environment is seen by some as a fatal flaw in Submarine Alkaline Hydrothermal Vent (AHV) theory; they instead point to wet-dry cycling in surface pools and ponds. However, as Ball reminded us, water is not only “life’s solvent”, but acts as a shell to, and interacts with, both proteins and their substrates [162]. Indeed, water is 70–80% of the cell mass, where it is deeply involved in the orchestration of cellular machinery [162,163,164,165,166,167,168,169,170,171]. Furthermore, all the dehydration-driven polymerization reactions of life occur in water. That they all require the mediation of protein machines—both to control the water activity at the active site, but also provide a means by which these inherently endergonic processes can be thermodynamically driven—merely highlights what the ‘mineral machines’ of ‘first life’ must also be capable of doing, and must be shown to be capable of doing (Figure 1 and Figure 2).

The rapidly precipitated micro- to nano-crystalline green rust and mackinawite comprising the alkaline mounds would also have included ambient water that was confined and somewhat immobilized through hydrogen bonding in galleries 0.3 nm deep between the brucite layers (e.g., in [Fe^II^_4_Fe^III^_2_(OH)_12_][CO_3_].3H_2_O). Thus, far from being a negative factor in the AHV theory for life’s emergence, the intimate polar interfacing with constitutional water in green rust is just one of the many common features that could link such minerals to emergent life [8,172,173]. Additionally, the anions required for charge parity in green rust were engaged through ion-dipole interactions with Fe^III^/Fe^II^ cations [20,88,89,90,103,151,152,158]. In strong contrast, nano-confinement of water in mackinawite greatly enhances its self-dissociation, incurring no entropic cost and permitting almost frictionless flow between the tetrahedral sheets [174]. Proton flow comparable to that in bulk water in these circumstances would have been via the Grotthuss mechanism at a rate of around 25 nm ns^−1^ [174,175,176,177,178]. This is one of the reasons we are now persuaded that it was the green rust and mackinawite micro- or nano-crysts and their associated interlayer galleries filled with structured or bulk water, respectively, that were life’s cellular precursors, rather than the fluid-filled chambers or gels that we, and others, originally imagined to have borne the first cytoplasm [5,30,45].

In the living cell, for example, water molecules hydrogen-bond to iron in heme and iron hydroxylase, to magnesium in the ribosome, and, for a pertinent organic example, water molecules stabilize and activate C-methyltransferase [179,180]. So, although the founding submarine alkaline mound was completely immersed in water, bulk water was excluded at the site of some reactions in the interlayers of green rust and mackinawite, just as it is by proteins or in lipid membranes. These are the minerals that not only provided the spontaneously deposited barrier that kept the two contrasting fluids from immediate mixing, but also, dosed with trace elements Ni, Zn, Co and Mo, they constitute the inventory in the minerals that could have acted as nano-engines and electron transfer agents (i.e., disequilibrium converters and free energy transformers) at life’s emergence [181,182,183,184,185,186,187,188,189,190,191,192,193]. In the following, we examine the main transfer agents—the layered micro- or nano-crysts: green rust and mackinawite. Notably, these can be produced in the lab with 10 mmol Fe(II) iron concentrations, well below the many tens of mmol of iron in the ~400 °C spring sources mentioned above (Figure 1 and Figure 2) [77,109,142]. We also touch on the likely, or possible, accompanying minor minerals: greigite, violarite, and tochilinite. We first consider the implication of a foundational presumption of the AHV theory: that life itself was driven into being by thermodynamic forces—as has every living thing since—by absolute necessity. What can we reasonably conjecture about those original forces that could have had those consequences?

## 5. The Disequilibria Imposed Across the Mineral Barrier

Given that life, in its bid to hydrogenate carbon dioxide and perhaps oxidize methane to a methyl group, obtains its power to do so from thermodynamically ‘downhill’ electron flow, it is reassuring to note demonstrations of electron generation sufficient to light light-emitting diode (LED) bulbs at both actual and experimental hydrothermal vents [74,75,76]. Whether electrons can be harvested directly from hydrothermal H_2_ across an abiotic mineral membrane or more substantial barrier is still uncertain. Other possibilities considered below are that they are either delivered (1) with formate, (2) through oxidation of methane by freshly precipitated Mo-dosed mackinawite or white rust, or (3) directly through the self-oxidation of micro or nano-crysts of these metastable minerals (Table 1) [15,19,66,67,73,74,75,76].

It is well established that the tensions between volcanic carbon dioxide against the hydrogen exhaling into the acidulous ocean of young, wet, and rocky planets cannot be released through mere geochemical reactions beyond formate due to thermodynamic barriers [184]. Even electrochemical experiments involving steep over-potentials do not produce the chemical intermediates that populate autotrophic metabolic pathways, such as methyl thioacetate (CH_3_COSCH_3_), which requires crossing the thermodynamic barrier posed by formaldehyde [34]. However, the endergonic reduction of carbon dioxide through formate to a formyl group is achieved in biology by coupling it to any one of three types of exergonic driving reactions: (1) the relaxation of a proton gradient, (2) the hydrolysis of polyphosphates, or (3) relaxing a redox gradient via electron bifurcation [24,27,31,78,92,94,98]. All these disequilibria conversions must be carried out by macromolecular nano-engines (disequilibrium converters) and, as we have intimated, we have every reason to assume that nano-engines were coopted from the minerals comprising the alkaline hydrothermal mound [4,17,40,159]. For reasons stated in Section 2, we look to hydrothermal methane to provide reduced carbon, along with formate, from the other end of the redox scale to the system. We now consider how this model corresponds to what is now known of the Hadean world.

The iron-rich mound generated at the alkaline vent offers an ambient trans-membrane proton motive disequilibrium or force (∆pH 4 to 5) as emphasized by Herschy et al. [24]. This mound also provides a redox gradient of ~500 mV comprising hydrogen plus methane versus nitrate and Fe^III^, Mn^IV^ plus carbon dioxide. At the same time, hydrothermal hydrogen at ~15 mmol/kg and formate at ~150 µmol/kg are born toward and focused at the mound [29,51,95,96]. Hydrogen is the *sine qua non* electron donor still used by all life in the form of NADH [194]. Indeed, through this hydrothermal process, billions upon billions of electrons are transported to the vent in hydrothermal solutions, exhaling at ~1000 kg/s in the present day ocean, enough to satisfy the most fuel-hungry microbial prototypes [195,196,197]. Temperature too was appropriate (30 ° to 100 °C), as was pressure (≥100 bars) and longevity (~10^20^ nanoseconds) [51]. The trace metals required for catalysis would be precipitated with the sulfides and oxides on meeting the margins of the alkaline vent fluid [16,17]. However, knowing how modern proteins work we have argued that endergonic reactions (a positive ∆G) cannot be forced to occur by mere catalysis, which can only reduce an activation energy[3,14]. So, catalysis alone cannot explain the emergence of life [4,7]. We need instead nanoscale disequilibrium converters situated at the margins of the alkaline hydrothermal mound to take advantage of the various gradients imposed by the precipitation of relatively insoluble inorganic entities. This seems to be a tall order: that one or more of such mineral micro- or nanocrystals might also act as the thermodynamic conversion engines driving emergent metabolism, in the sense of Cottrell, seems counter intuitive, viz., could the local redox-state effects on gallery conformation, especially in green rust, drive the needed proto-biosynthetic reactions while retaining their overall integrity [181,182,183]?

We hypothesize that the proton and electron gradients, as in life, have the theoretical potential to drive endergonic reactions toward a break-out metabolism [16,17,18,54]. For one, the disequilibrium between the redox couples H^+^/H_2_ and NO_3_^−^/NO_2_^−^ of ~800 millivolts has also now been well established [57,198]. And if the electrons bifurcated—one to the high potential acceptor nitrate—the other could have provided the over-potential sufficient to have driven, for example, the reduction of carbon dioxide. In addition, the proton gradient was steep. The pH of the Hadean ocean likely oscillated around five pH units given the continual escape of CO_2_ from the so-called “popping rocks” comprising the ubiquitous submarine mafic lava flows, and the empirical values reported from Lakes Nyos and Monoun in the Cameroon, as against the alkaline (pH ~11) hydrothermal solution, producing a gradient of 250 to 300 millivolts (one pH unit being equivalent to 59 mV) [30,144,145]. Thus, the exact pH disequilibrium is not critical beyond offering a potential of ~200 mV.

It should be noted here that ferrous carbonate remains super-saturated in these conditions [83]. And the fact that both the oxyhydroxides and the sulfides can house substantial nickel concentrations in structures affine with active sites in the metalloproteins makes them promising promotors for the disequilibrium converters (free energy transducers), or nanomotors and electron transfer agents (protoenzymes) at life’s emergence. However, the big question remains as to how these disequilibria were coupled to surmount those endergonic reactions along the pathways to metabolism.

Of course, a detailed research effort is required to reveal how protonic and electronic disequilibria could have been converted abiotically, presumably by the minerals discussed above, to function as theorized (Figure 2). One obstacle to understanding how exergonic processes were able to drive endergonic ones is quite counterintuitive [14]. For example, such driving does not involve the transfer of energy (even the free part of a literal energy) from the driving process to the driven one [14,182]. Rather, the engine managing the conversion causes individual fluctuation events that dissipate the driving reaction contingent on the coincident occurrence of an endergonic (reverse) fluctuation in another thermodynamically weaker process, thereby creating a disequilibrium in that process [3,7,14,199,200].

## 6. Green Rust—The First Organizing Nanoengine of Life?

Carbonate green rust (GR_carb_) nucleates rapidly to produce a plethora of equi-dimensional planar micro- to nano-crysts, generally measuring from 200 nm to ~2 µm across, with a relatively constant aspect ratio of ~1:10 [152,201,202]. So, a 1-µm GR_carb_ would be about 100 nm thick and comprise ~130 double layers [201,202,203,204,205]. The height of the hydroxyl interlayer galleries is ~0.30 nm, allowing them to perhaps act as anion and proton channels. The Fe^2+^-Fe^3+^ distance of ~0.33 nm along the iron layers would allow electron hopping by quantum tunneling, though a small proportion of electrons could also jump across the layers [19,20]. The brucite layers trap a monomolecular interlayer of viscous solution producing solutions crowded with simple anions to concentrations of up to 14 molar [8,206].

Green rust is redox active and can rapidly reduce nitrate to the ammonium ion as well as the redox-sensitive ions like Mo^VI^ to Mo^IV^ [86,87,88,89,90,207,208,209,210,211,212,213]. Complexes of such redox-sensitive trace metal ions tend to be reduced and precipitated from their soluble source at, and as, the outer edges of green rust are oxidized [210]. Such metastability would threaten the integrity of GRs were it not for their partially oxidized margins being highly attractive to orthophosphate (HPO_4_^2−^). The ionic diameter of the phosphate ion at 0.48 nm, considerably exceeding those of the interlayer galleries at ~0.3 nm, causes it to block the GR margins and inhibit its oxidation [203,204,205,206]. However, orthophosphate (Pi) has also been demonstrated to intercalate into hydrotalcite where it raises the normal gallery height to 0.37 nm [214,215]. We speculate that protons ramming from the acidulous ocean would drive a portion of this phosphate into the hydrous interlayers of the green rust DLH[4,18,214,215]. Once there, it could be forced to polymerize—the external proton gradient driving a strong pyrophosphate/orthophosphate (PPi:Pi) disequilibrium [18]. Phosphorylation of organic products of redox reactions involving carbon might then ensue [18]. The resulting breaks in the phosphate envelope to the green rust would be instantly healed as more phosphate is attracted to the positively charged exterior[216].

To make green rust work as a thermodynamic engine to drive reductions, it would itself have to be continuously re-reduced. In this regard, green rust has also been demonstrated to be reduced by lactate and hydrocarbons without degrading its structure, but whether methane could also reduce relatively oxidized green rust (via activation of NO), as we have suggested, must await experimental testing [54,55,198,217,218,219]. Another possible, though undemonstrated, method would be the oxidation of hydrothermal hydrogen with nickel-dosed mackinawite or greigite, as in a Ni-Fe hydrogenase [40,64,66,220,221]. The resulting protons would be immediately neutralized by reaction with the hydroxyls in the alkaline hydrothermal flux. In other words, it is reasonable to assume that the redox states could be reversed (oscillate) depending on the stacking of the disequilibria. Green rust has a low redox potential, being almost as reducing as native iron (Fe^0^) in anoxic alkaline conditions [222]. Indeed, as we have noted, GRs can compete with biotic pathways in the reduction of, for example, selenate and nitrate [222]. Moreover, we have seen how green rust, with its well-ordered hydrous innards, can act as an aminase. For example, Barge et al. also demonstrated the amination of pyruvate to alanine [39].

There is an interdependence between the redox potential and the pH of green rusts: oxidation is promoted by deprotonation in alkaline conditions, whereas the reverse is true of acid conditions where protonation supports reduction [19]. These factors prompted Génin et al. to remark, “Among all compounds containing Fe(II) ions that can play the reservoir of reducing species, the Fe(II)−(III) oxyhydroxysalts commonly denominated green rusts … constitute an unmatchable compound due to their redox flexibility” [90]. As might be expected, solid state reductions of oxidized green rusts can also be driven electrochemically, resulting again in a highly reduced but still morphologically recognizable double layer hydroxide ([Fe^II^_6_(OH)_10_][CO_3_.2H_2_O]) (Figure 2) [90,152,223]. Nevertheless, without the protection of organic intercalates, there is a tendency for the Fe^2+^ to dissolve, thereby threatening the integrity of the mineral [110,112,222,223].

Although these green rust-comprising barriers would have been leaky, individual crystallites would have hampered immediate dissipation [30]. Green rusts comprising this barrier would be subject to large redox and pH gradients with ranges that had the potential commensurate with those required for the onset of metabolism. Electrons could have been supplied from hydrothermal formate, hydrogen, or from oxyhydroxides and sulfides, dosed with other transition metals comprising the walls of hydrothermal chimneys as they oxidize [17,64,73,74,75,76]. Redox oscillations may have oxidized and re-reduced iron ions between Fe^2+^ and Fe^3+^, much as our hemoglobin is cycled between zero and four oxygens many times over as the red cells are pumped from the lung to the periphery and back with a cycling time of around one minute and with an average life span of 1000 to 2000 h. At the same time, the pH gradient would have contributed to protonation and deprotonation of green rust, tending to hold the mineral between the bounds of 2Fe^2+^/Fe^3+^ and Fe^2+^/2Fe^3+^ [19,211,224]. The mineral so poised would have remained metastable, i.e., could have been party to an emergent homeostasis [19,225]. Such metastability may have been strongly supported by orthophosphate (HPO_4_^2−^), which, having a larger ionic diameter of 0.48 nm, tends to be adsorbed on the margins of green rust and inhibits its oxidation [223]. As we have noted, Arrhenius extolled the likely prebiotic virtues of the double layer hydroxides, saying:

Surface-active DLH minerals expand freely to accommodate molecular complexes of any size. These structures, thus, also serve as compartmental systems with flexible membranes and what may be called primitive cellular metabolic function. Like cells, they retain phosphate-charged reactants against high concentration gradients and exchange matter with the surroundings by controlled diffusion through the ‘pores’ provided by the opening of the interlayers at the crystal edges. Here, the exposed negative charge on the interrupted metal hydroxide ‘membrane’ leads to sorption of cations as ‘gatekeepers’.

[8]

He even goes on to speculate that the origin of information might lie in “the variable pattern of cations, conserved within individual (DLH) crystals,” and further that “such an animation process … would be guided by the subtle electronic forces that metal cations exert on their environment,” while opining that “the progress so far in prebiotic chemistry merely suggests that it may be possible to synthesize the tape on which the information for life could be recorded, if we only knew how [8].”

The margins of green rust would be continually ‘rammed’ by hydronium ions from the ~5 pH ocean as the protons themselves were drawn down-gradient to the pH 11 interior [4]. Their path would be vectored by the natural orientation of these layered minerals normal to the barrier’s surfaces with the hydrous interlayers acting as channels [45,112]. Additionally, it may have been within the confines of green rust at high pH that condensation reactions could have been driven. Erastova et al.—noting how mineral surfaces “may have very high enthalpies of rehydration” and thereby provide “a driving force for condensation reactions”—have suggested, through molecular dynamics simulations, how layered hydroxides can concentrate, align, and act as adsorption templates for amino acids and promote peptide bond formation [103]. Moreover, following Amend and Shock, Kitadai found that—in regard to the thermodynamics of amino acid synthesis and polymerization—“hot, alkaline hydrothermal systems beneath cool, slightly acidic Hadean ocean are an energetically excellent setting among possible vent-ocean combinations” [226,227].

It might be argued that the water generated in these protobiochemical processes would tend to lyse any product. However, we argue that because the founding submarine alkaline mound was completely immersed in salty water both inside and out, bulk-water itself was minimized at the site of these and other reactions as a result of exosmosis from the hydrous interlayers, thus obviating any supposed need for wetting and drying cycles (Figure 2).

## 7. Iron Sulfides: The Supporting Cast

### 7.1. Mackinawite [Fe(Ni,Mo)S]: Electron Transfer Agent, Amino Acid Polymerase, and Possible Hydrogenase

Mackinawite has an extremely low redox potential of −1010 mV at pH 12 at 25 °C, lower even than green rust [228,229]. In addition, 20% of the total iron in mackinawite can be oxidized before its layered conformation is lost and converts to the water-absent inverse spinel, greigite (Fe_3_S_4_) [229,230]. However, both nickel concentrations and the infusion of molecular H_2_ can inhibit this transition [64,69,70,231]. Indeed, up to ~50% of the iron can be replaced by nickel without changing the mackinawite conformation, although the solid solution is non-ideal [70]. Notably, the Mo:Fe molar ratios in mackinawite precipitated in the presence of thiomolybdate can reach ~0.05 [71]. Both nickel and molybdenum are known catalytic promotors [80,232].

Unlike bound water in green rust, interlayer-water in mackinawite is only lightly bound; nanoconfinement of water greatly enhances its self-dissociation. This is because the effective viscosity therein is similar to that of bulk water—strongly favoring the rapid diffusion of excess protons via the Grotthuss mechanism [177,178]. Mackinawite is also known to be an excellent conductor, suggesting that electron-hopping along the iron layers in mackinawite could be coupled to proton transfer using intermolecular hydrogen-bonds through fluctuation and sequential H-bonding, as observed in peptides [21,231,233]. Such coupling of proton flux to electron flow, and vice-versa, would be comparable to the proton-coupled electron transfer (PCET) reduction of nitrite to NO reaction involving cytochrome cd_1_ in nitrite reductase [79,176,234,235]. Indeed, we can imagine the aqueous interlayers within mackinawite acting as a proton wire with a rate of proton transfer over 25 nm ns^−1^ (about a 10th of the speed of sound) driven by a redox disequilibrium of ~1 eV [174,175,236]. Yet the hydroxyl ions (or proton holes) tend to be immobilized by hydrogen-bonding to sulfur and the dangling oxygen is partially hydrated, thus inhibiting the diffusion of hydroxyls through the galleries from the alkaline hydrothermal fluid, and thereby favoring proton transfer [174,236].

Another biotic-like feature of these nano-confined conditions between the tetrahedral FeS sheets of mackinawite micro-crysts is suggested by the nano-solvation of glycine and its polymerization in the presence of carbonyl sulfide (Table 1) [104,108].

### 7.2. Greigite [Fe_3_S_4_]

In the experimental growth of iron sulfide chimneys White et al. found that mackinawite was partially oxidized to greigite [Fe^2+^Fe^3+^_2_S_4_] between 70 and 75 °C [64]. We assume, after Rickard and Luther, that the reaction is autocatalytic with water as the oxidant [237]. The hydrogen produced in this reaction maybe occluded or trapped, perhaps in the cuboidal entity of the greigite structure where it may be split, with electrons delocalized such that the average charge on the metals is decreased in the cuboid while the sulfides are protonated [16]. The sulfur atoms would remain in place during the transition, though the mackinawite *a* axis is at a 45° angle to that of greigite [238,239].

Although the structure of greigite is afine with the active centers of ferredoxins (e.g., in molybdenum-bearing formate hydrogenlyase and radical SAM, and is comparable to the cuboids in the active centers of carbon monoxide dehydrogenase and acetyl coenzyme synthase, no passive catalytic activity in these minerals has (yet) been demonstrated [240,241,242,243]). Micro-crysts of greigite are magnetic and have low electrochemical resistance [244,245,246]. Greigite is an anhydrous inverse spinel wherein the apical iron (or nickel) is in the 2^+^ state, whereas the delocalized electrons produce an average charge of 2.5^+^ on the four iron ions in the cuboid [16].

Nickel can deputize for iron in greigite, generally in ~Fe_5_NiS_8_ or as violarite ~Fe_2_Ni_4_S_8_ [34,212]. Yamaguchi et al. demonstrated the electrochemical reduction of carbon dioxide to formate and methane by coating a carbon paper electrode with violarite [34]. Efficiency, as we might expect, was substantially increased with addition of amine compounds [34]. Yet, in contrast to its occlusion in mackinawite, molybdenum appears to be excluded from the inverse spinel, though Ni-bearing greigite can be coated with layered MoS_2_ in the lab [247]. Dosed thus, Sharifvaghefi and Zheng showed that it can promote both hydrogenation and desulfurization reactions of, for example, dibenzothiophene (DBT, H_4_C_6_S C_6_H_4_), while remaining stable [247].

### 7.3. Tochilinite FeS[Mg,Fe^2+^][OH]_2_

We note here that though never identified as a precipitate in the AHV conditions, mixed hydroxide and sulfide minerals, tochilinite (FeS[Mg,Fe^2+^][OH]_2_) and ferrotochilinite (6FeS·5Fe(OH)_2_), could also have been represented in the hydrothermal mound where they might have played a part in early metabolism [248,249,250]. These composite minerals comprise six mackinawite-like layers alternating with five brucite-like layers [251]. They can be grown below 200 °C in alkaline hydrothermal solution, but as a result of the two-dimensional incommensurability of their sub-structures, they tend to curl into nanotubes [252]. Tochilinite occurs in serpentinized mafic and ultramafic rocks and in CM carbonaceous chondrites, where it appears to result from moderate temperature hydrothermal alteration of olivine- and sulfide-rich precursors [251,252,253,254,255,256,257].

## 8. The Peptide and Amyloid Takeover?

Barge et al. demonstrated the amination of pyruvate to alanine in alkaline conditions within moderately reduced green rust [39]. Nevertheless, amino acids have not been polymerized in green rust to date. However, short peptides have been generated in conditions corresponding to the alkaline vent environment [227]. Once formed, even six-mer peptides spontaneously and promiscuously sequester inorganic Fe(Ni)sulfides, metal ions, and pyrophosphate (PPi) clusters [36,37,114,115,116,117,118]. Such spontaneous folds around phosphate survive today as the GxxxxGKS/T motif of P-loop proteins [258,259,260]. The achiral glycines contribute the universal joints required for this coordination, generally referred to as the Walker motif—possibly a reminder of the emergence of an heterochiral world before the left-handed chirality of peptides became dominant [261,262].

As it is the backbone of amide hydrogen bonds that coordinate the clusters, in these early times, the side chains of amino acids played little part beyond perhaps merely binding two essential metal ions and orienting substrates in the so-called “two-metal-ion catalytic site” [263,264,265]. The nesting itself renders the clusters more stable, more catalytically active, and in the case of the sulfides, significantly reduces their redox potential. These properties may have both quickened and directed the early evolution of metabolism. Indeed, these are the structures that were locked into many of the proteins of life as they evolved and complexified.

Many oligopeptides whose residues are sufficiently hydrophobic, aggregate in water into amyloid fibrils. These structures stabilize the oligopeptides against proteolysis and can be capable of conformational self-replication. For example, oligopeptides comprising ala-ile-leu-ser-ser-thr pack as antiparallel beta-sheets that, in spite of having a dry hydrophobic core, can offer water-mediated interfaces, cf. the aquaporin channel [117,266,267,268,269]. Greenwald and Reik speculated that this was the common ancestor fold [268]. The sheets are capable of self-propagation, carrying information, and can be conformationally self-replicating [269,270,271,272,273]. Amyloid has also been suggested by many as the likely first organic cell wall and membrane, favored over the leaky and inefficient iron mineral barriers [117,274]. Superior to lipids, which are ‘costly’ to make, suffer the disadvantage of impermeability, so likely evolved later; they maintain the capability of proto-enzymetic activity [92,117,261,273,274,275,276].

## 9. Ligand-Assisted Autocatalysis and ‘Protoenzymes’ 

Five- to six-mer alanine nests, sequestering anionic clusters of the iron oxyhydroxides and sulfides, could also have played an essential role in mineral redox-powered disequilibria-converting engines driving ligand-accelerated autocatalytic reactions. Through this process, geo-mechanochemical reactions could have been driven along the mineral-enabled pathways toward methanotrophy and acetogenesis, ever improving as peptides sequestered active clusters, eventuating in an incomplete reverse tricarboxylic acid cycle before biochemistry proper took over. Table 1 indicates the possible steps in the putative ligand-accelerated pathway to life’s emergence, and Figure 1 details the possible transition from the earth to life sciences.

## 10. Emergence of Life as a Biofilm

We suggested here that amyloid-forming peptides would have been the first organic polymers to be produced within the interlayers of green rust and that these would be secreted to envelop their layered iron mineral parents (Figure 2). We further speculate that pods of amyloid-bearing micro- or nano-crysts and peptide nests may have differentiated to protocells of a common size dictated by water content and binding forces. Such cells may have developed different functions, perhaps forming a symbiotic cooperative. In this scenario, we might imagine the intercellular spaces being occupied by amyloid binding the individual proto-cells together to create individuated cellular cooperatives [277]. Thus, the first palpable living communities, still at the hydrothermal mound, could have been biofilms. Adherence, then as it is often now, provided by excess amyloid, was probably augmented with carboxylates [278,279,280,281,282,283,284,285]. These putative biofilms comprising minimal organic cells may have resembled iron-bearing microbialites [286].

## 11. Discussion of Method and Approach

We began this article with a quote from Leduc as a basis for the quest to understand life’s emergence [1]. We quote him again here to give context for this unending quest and to remind us that some provisional explanation is required for the emergence of life to give root to the Darwinian program [287]. Leduc insists that:

The chain of life is of necessity a continuous one, from the mineral at one end to the most complicated organism at the other. We cannot allow that it is broken at any point, or that there is a link missing between animate and inanimate nature (viz. the missing link between the inorganic and the organic kingdoms). Hence the theory of evolution necessarily admits the physico-chemical nature of life and the fact of spontaneous generation. Only thus can the evolutionary theory become a rational one, a stimulating and fertile inspirer of research.

[1]

Or more bluntly “Without the idea of spontaneous generation and a physical theory of life, the doctrine of evolution is a mutilated hypothesis without unity or cohesion [1].”

Darwin was certainly conscious of the need to understand life’s emergence to provide the root for his evolutionary tree but considered the issue beyond the confines of science, as was true then [288,289,290]. Indeed, in a letter to Hooker in 1863, he concluded with:

Who would have ever thought of the old stupid Athenæum taking to Oken-like transcendental philosophy written in Owenian style! It will be some time before we see “slime, snot, or protoplasm” (what an elegant writer) generating a new animal. But I have long regretted that I truckled to public opinion and used the Pentateuchal term of creation, by which I really meant “appeared” by some wholly unknown process. It is mere rubbish thinking, at present, of origin of life; one might as well think of origin of matter.

(https://www.darwinproject.ac.uk/letter/DCP-LETT-4065.xml)

It is often overlooked that Darwin’s approach was steeped in the geological milieu he shared with his mentors, Adam Sedgwick and Charles Lyell, two of the founders of modern geology. Indeed, he was a self-proclaimed geologist himself, so it is all the more surprising that earth science is given such short shrift by chemists who rarely consider the initial conditions offered to them by geology [289,290]. To only report experiments that happen to work in the lab, that seem to mimic certain of life’s pathways, but using unverifiable, indeed, implausible, prebiotic ferrocyanide feedstocks, leads us to wander into a miasma of uncertainties. It is time to reflect on Polanyi’s admonition, “It is only when we are confronted with the anxious dilemma of a live scientific issue, that the ambiguity of the formal processes and of various attenuated criteria of scientific truth becomes apparent, and leaves us without effective guidance” [291]. In other words, we have to think widely and beyond our disciplinary boundaries [291,292].

It is true that the notion of spontaneous generation had fallen into disfavor, where it still resides, since Pasteur’s famous experiment [293]. Darwin himself sought to distance himself from the idea [288]. Indeed, Butcher recounts how Pasteur’s acolytes prevented publication of Leduc’s hypotheses for some time in France—hypotheses that remain disparaged even in this new century [1,294]. However, it should be remembered that the phrase tends to be understood to support a system supplied with the organic building blocks of life, whereas Leduc’s notion assumed the spontaneous generation to have been in an inorganic milieu [1]. The AHV theory reiterated here also assumes a spontaneous inorganic emergence of life—a theory yet considered inconsistent with advances made in the laboratory [295]. Again, this is to misunderstand how completely new knowledge is attained [291,292]. The outcomes of theory and experiments should lead toward palpable goals. In this regard, the AHV theory did predict the presence of off-ridge submarine alkaline vents in the present oceans, a prognosis met by the discovery of the Lost City submarine alkaline vents in 2000 [294,295]. It also explains, for example, why early life did not have to invent such a counterintuitive mechanism as that entailed in Mitchell’s proton motive force, how it was supplied with the necessary low entropy C1 feed, how biosynthesis could proceed in a highly radiated and neutral atmosphere, and why it was not destroyed by surface catastrophes in the Hadean[3,4,16,18]. Here, we not only list experiments favoring the theory overlooked by, for example, Ritson and his coworkers, but detail further predictions, some of which could be falsified, reveal embarrassing missing links, or even leave the AHVT as just one more casualty of the general theory of natural rejection (Table 1) [296,297]! In Feynman’s exacting dictum, here, “we are trying to prove ourselves wrong as quickly as possible, because only in that way can we find progress” [298].

## 12. Conclusions

The purpose of this contribution was to suggest how the submarine alkaline hydrothermal vent model could be subjected to stringent tests that would indicate its failure, or partial failure, to provide a path forward in emergence-of-life research. The suggestions detailed in Figure 1 and Table 1, built on some empirical evidence, assume that the 150 or so hydrous interlayers of green rust, clamped between layered pliable redox-active iron hydroxide boundaries dosed with Ni, Co, and Mo, and supported by iron sulfides, could have provided the potential to: (1) differentiate and specialize functions such as proton-pumping and thereby the generation of a far-out-of-equilibrium PPi:Pi ratio, (2) enable electron bifurcation, (3) reduce CO_2_ to formate or carbon monoxide, (4) oxidize hydrogen and methane to methyl groups to react with formate or CO and thereby, (5) produce acetate and pyruvate, (6) reduce nitrate, (7) aminate carboxylic acids to the simple amino acids, and (8) polymerize these acids to heterochiral peptides to protect the evolving system at its various scales. All-in-all, these processes could have resulted in the germination and first flowering of the organic evolutionary tree as it emerged from the hydroponically-fertilized green rust seed. The activity of the water generated in these proto-biosynthetic reactions may have been kept low in the hydrous innards of green rust and mackinawite through exosmosis to the salty surrounds [59,103].

The AHV theory as it stands focuses on how the hydrous interlayers or channels in green rust, and to a lesser degree in mackinawite, likely mediated the imposed proton gradient and, through the bifurcation of electron pairs, effectively stepped up the redox gradients to drive an organic takeover [4]. We are conscious that the relative contributions of green rust, mackinawite, and associated minerals, and how they may have cooperated in Leduc’s synthetic biology, has been left unresolved. We alert the reader to a significant revision of the AHV theory since its first proposal, namely that although we still recognize that the job of life overall is to hydrogenate carbon dioxide, it may be that life first captured both the partially and fully reduced forms of C1 carbon as hydrothermal formate and methane. Only later (though well before LUCA) did life ‘learn’ to reduce CO_2_ through all the required intermediates for CO_2_ autotrophy to emerge from its mineral placenta [50,54,55].

At base is the assumption that the protonically- and electronically-powered nano-engines and disequilibrium conversions needed to drive those endergonic reactions, required to produce life’s many processors and its superstructures today, were initially coopted from iron oxyhydroxides and sulfides, dosed with transition metals and phosphate, precipitated at the submarine alkaline vent [6,17,30,299]. However, even in the light of some experimental support, we can see no clear path to the nucleotide world and, beyond intriguing suggestions of Greenwell and Coveney and others, the theory dissipates in hypotheses, ideas, and speculation [8,34,300]. Nevertheless, “that living organisms are distinguished, not by their momentary appearance, but by their behavior and by their relationship to their environment” in Mitchell’s memorable phrase, would also be true for the putative layered hydrated seed crystals to life [301]

So, maybe it all began with the selection of thermodynamically-driven micro-crysts that could improve (increase the system’s dissipative flow) by becoming more organized and complicated, and/or by growing or declining in size, and/or by replicating. So, in doing any or all of these more efficiently, they could have captured the driving disequilibria to convert those disequilibria ever faster and more efficiently on the path to subjugation by the organic world. However, showing how selection for the fittest in this sense can happen, or does happen, in mineral precipitates remains a major challenge [3,4,55,221].

## Figures and Tables

**Table 1:** Steps toward ligand-accelerated autocatalytic denitrifying methanotrophic acetogenesis: demonstrated, analogous, probable, possible, and predicted, with comparisons to enzymes.

Biosyntonically ‘Engineered’ Steps,		Mineral Barriers, Engines, Catalysts	Abiotic Reaction Coupling and/or Gradient	References
cf. Prebiotic Enzyme Analogues				
0. {5OH^−^ + HS^−^} + 2Fe^2+^ + Fe^3+^ + Ni^2+^ → {FeS + Fe_2_(OH)_5_} + ē		Green rust and [FeNi]S set in SiO_2_?	Spontaneous barrier precipitation	[6,22,24,30,31,35,45,59,65,67]
**membrane**				
1. H_2_ → 2H• → 2H^+^ + 2e^−^		GR>FeS>NiS>MoS_2_/Chimneys	Redox gradient	[68,69,70,71,72,73,74,75,76,77,78,79]
**NiFe[Mo]-Hydrogenasase**				
1a. proton-coupled electron transfer processes		GR, mackinawite, greigite	Proton gradient	[79]
**Ferredoxin**				
1b. electron bifurcation, conformation plasticity, electron and proton transfer, gating	**MMO**, **Nir**	GR, mackinawite, greigite, MoS_x_	Redox gradient	[4,7,8,18,19,20,61,67,72,73,74,75,76,77,78,79,80]
2. H^+^ + 2ē + CO_2_ → HCOO^−^ + H_2_O	**FHL**	Ni_3_Fe, or [FeNi]S or MoS	Serpentinization, or redox, pH gradient	[24,60]
2a. CO_2_ + 2ē + H^+^ → CO + OH^−^	**CODH**	Violarite	Electron conduction	[34,81,82,83]
3. CH_4_ + NO_3_^−^ + H_2_ + H^+^ → •CH_3_ + 2H_2_O + NO		GR & Mo-dosed greigite (redox/pH gradients)	Undemonstrated (Redox and pH gradient)	[54,55,61,80,84,85]
**MMO**				
4. NO_3_^−^ + 4H_2_ + 2H^+^ → NH_4_^+^ + 3H_2_O		GR (redox/pH gradients)	Redox (~180 min)	[86,87,88,89,90,91]
**Nar/Nir/NOR**				
5. •CH_3_ + OH^−^/SH^−^?) → CH_3_OH/ CH_3_SH) + ē		GR? high T	Low yield	[43,44]
**MMO?**				
6. CH_3_OH + [2Fe^III^] → HCHO + [2Fe^II^] + 2H^+^	**MDH**	GR [FeNi]S? Fe_2_(MoO_4_)_3_	Undemonstrated	[54]
7. HCHO+HP_2_O_7_^3−^ + [OH^−^] → [HCOPO_4_]^2−^ +HPO_4_^2−^		?	Undemonstrated (exergonic)	[92]
**FK**				
8. HCOPO_4_^2−^ + HS^−^ + 2H^+^ + 2ē → CH_3_S^−^ + HPO_4_^2−^		?	Undemonstrated (exergonic)	[92,93]
9. CH_3_S^−^ + HCOO^−^ + H^+^ → CH_3_COOH + HS^−^		Fe_4_NiS_9_(HN)_2_	cf. Reppe chemistry	[29,42,92,93,94,95,96]
**ACS**				
9a. CH_3_S^−^ + CO → CH_3_COS^−^		Fe_4_NiS_9_(HN)_2_	High yield (20 h)	[42]
9b. (CH_3_COS^−^ + HPO_4_^2−^ → CH_3_COPO_4_^2−^ + HS^−^)		?	Low yield	[94]
10. HCOO^−^ + CH_3_CO~SCH_3_ + ē → CH_3_COCOO^−^ + HSCH_3_^−^		Fe_2_(RS)_2_(CO)_6_	Undemonstrated	[97,98]
**PFL**				
10a. CH_3_COCOO^−^ + (HP_2_O_7_)^3−^ + CO_2_ → CH_2_C=C(O**P**O_3_)^2−^COO^−^ + HPO_4_^2−^ +H^+^		GR/mackinawite?	Predicted	[16]
**PPase**				
10b. CH_2_=C(OPO_3_)^2−^COO^−^ + CO_2_ + H_2_O → ^−^OOCCH_2_COCOO^−^ + HPO_4_^2−^ + H^+^	**ACC**	GR/mackinawite?	Predicted	[16]
11. CH_3_COCOO^−^ + NH_4_^+^ + 2ē + 2H^+^ → CH_3_CH(NH_2_)COO^−^ + H_2_O	**ALT**	GR/mackinawite?	24 h	[39]
12. (CH_3_CH(NH_2_)COOH)_4_ + CH_3_CH(NH_2_)COOH → CH_3_CH(NH_2_)CO-CH_3_CH(NH)CO-CH_3_CH(NH)CO-CH_3_CH(NH)CO-CH_3_CH(NH)COOH + 4H_2_O		Dolomite (*ab initio* simulations mackinawite and double layer hydroxide)	Spontaneous (Dolomite)	[99,100,101,102,103,104,105,106,107,108]
**DLH [cf. “DNA pol”]**				
13. Pi + Pi → PPi by GR	**H^+^-PPase**	FeS, GR	Only at ~equilibrium	[109]
**ligand-assisted** **recapitulation?**				
{13} poly-alanine peptide-strengthened **membrane**?		mineral-organic framework	Spontaneous	[65,110,111,112]
{14} SGAGKT peptide + Pi →	**P-loop**	6mer peptide	Spontaneous	[113]
{15} CH_3_CH(NH_2_)CO-CH_3_CH(NH)CO-CH_3_CH(NH_2_)CO-CH_3_CH(NH)COOH + Ni^2+^ → Ni-CH_3_CH(NH_2_)CO-CH_3_CH(NH)CO-CH_3_CH(NH)CO-CH_3_CH(NH)COOH	**ATCUN motif**	4mer peptide	Spontaneous	[94,95,96,97,98,99,100,101,102,103,104,105,106,107] [114,115,116]
{16} (Fe_4_NiS) + CH_3_CH(NH_2_)CO-CH_3_CH(NH)CO-CH_3_CH(NH)CO-CH_3_CH(NH)CO-CH_3_CH(NH)COOH → [Fe_4_NiS]-CH_3_CH(NH_2_)CO-CH_3_CH(NH)CO-CH_3_CH(NH)CO-CH_3_CH(NH)CO-CH_3_CH(NH)COOH	**Proto-fd**, **ACS**, **CODH**	6mer peptide	Partial demonstration	[117,118]
→ {16}{1}{2}{3}{4}{5}{6}{7}{8}{9}{10}{11}{12}{13} → repeat		GR breakout metabolism?		Figure 1

ACC = Acetyl-CoA carboxylase; ACS = Acetyl-CoA synthase; ALT = Alanine transaminase; ATCUN motif = Amino terminal Cu(II) and Ni(II) binding motif; CODH = Carbon monoxide dehydrogenase; (DLH = Double Layer Hydroxide; DNA pol = DNA polymerase); Fd = Ferredoxin; FHL = Formate hydrogen lyase; FK = Formate kinase; H^+^-PPase = proton pyrophosphatase; MMO = methane monooxygenase; MDH = methanol dehydrogenase; Nar = nitrate reductase; Nir = nitrite reductase; NOR = nitric oxide reductase; NiFe[Mo]-H_2_ase = NiFe[Mo]-hydrogenase; PFL = Pyruvate formate lyase; ? = uncertain.
